# Lipopolysaccharide Sensitizes Steroid-Induced Brain Injury in Neonatal Rat Pups

**DOI:** 10.1155/mi/8285898

**Published:** 2025-11-20

**Authors:** Yu-Shan Chang, Tzu-Mo Yang, Yu-Ling Hsu, Yu-Min Kuo, Chyi-Her Lin

**Affiliations:** ^1^Department of Emergency Medicine, Chi Mei Medical Center, Tainan, Taiwan; ^2^Department of Cell Biology and Anatomy, College of Medicine, National Cheng Kung University, Tainan, Taiwan; ^3^Department of Biotechnology and Bioindustry Sciences, College of Bioscience and Biotechnology, National Cheng Kung University, Tainan, Taiwan; ^4^Department of Pediatrics, E-Da Hospital, I-Shou University, Kaohsiung, Taiwan; ^5^Department of Pediatrics, College of Medicine, National Cheng Kung University, Tainan, Taiwan

**Keywords:** brain injuries, cerebellum, dexamethasone, hydrocortisone, lipopolysaccharides

## Abstract

**Background:**

Infection is a pathogenetic factor for bronchopulmonary dysplasia (BPD), and corticosteroids are often used for its prevention or treatment. However, few studies have examined their combined effects on brain injury in the context of infection.

**Methods:**

Rat pups received lipopolysaccharide (LPS) on postnatal Day 1 (P1), followed by tapering doses of dexamethasone (Dex) or hydrocortisone (HC) from P2 to P4. We measured body and brain weights, TUNEL-positive cell counts, synaptic protein levels, and mRNA expression of glucocorticoid receptor (GR) and mineralocorticoid receptor (MR) in six brain regions at P5.

**Results:**

The LPS-HC and LPS-Dex groups showed more TUNEL-positive cells in the hippocampus, cerebellum, and brain stem compared to LPS-naïve controls. Oligodendrocyte precursor cells were the predominant TUNEL-positive cells in the hippocampus and brain stem. Additionally, the LPS-Dex or LPS-HC group showed significantly reduced levels of postsynaptic density protein 95 (PSD95), a postsynaptic protein, in these regions, while treatment with Dex or HC alone did not impact PSD95 expression. GR mRNA was significantly reduced in cortex, striatum, hippocampus, and cerebellum in LPS-HC group, with MR mRNA reduction limited primarily to the striatum.

**Conclusions:**

LPS sensitized the immature brain to Dex or HC-related cell death to possible apoptosis and augmented the LPS-induced disruption of synaptic integrity in certain brain regions, potentially via altered GR and MR expression that may modulate corticosteroid receptor signaling.

## 1. Introduction

Bronchopulmonary dysplasia (BPD) remains one of the greatest challenges in neonatology [1]. Inflammation plays a central role in the pathogenesis of BPD and could be evoked by infectious organisms, oxidative stress, or mechanical ventilation. As cautious use of oxygen or noninvasive ventilation is becoming the standard of care for very preterm infants, sources of inflammation other than oxidative stress and mechanical injuries, such as chorioamnionitis or postnatal infection, are becoming increasingly important as pathogenetic factors for BPD [2, 3].

Postnatal corticosteroids remain one of the few effective strategies for the prevention or treatment of BPD in preterm infants. The European consensus guidelines on managing respiratory distress syndrome recommended a short tapering course of low-dose dexamethasone (Dex) to facilitate extubation in infants who remain on mechanical ventilation after 1–2 weeks of age [4–6]. Hydrocortisone (HC) is increasingly being used as an alternative to Dex in clinical practice. The 2022 AAP guideline on the use of postnatal corticosteroids further specified that early (<7 days) low-dose HC may prevent BPD or death in infants weighing less than 1000 g and exposed to chorioamnionitis. Animal studies suggest HC may be a safer alternative [7, 8], and clinical trials indicate that prophylactic HC in extremely preterm infants were not associated with adverse neurodevelopmental outcomes up to 5 years of age [9, 10]. The magnetic resonance imaging (MRI) examinations of the brain also did not find significant lesions after HC exposure [11, 12].

Fetal or neonatal lipopolysaccharide (LPS) exposure causes white matter brain injury resembling cerebral palsy in animal models [13, 14]. Retrospective studies in humans also identify chorioamnionitis as an independent risk factor for cerebral palsy in term and near-term infants [15]. and its presence in the placenta is linked to adverse neurological outcomes in neonatal encephalopathy [16]. In a fetal sheep model, antenatal glucocorticoids aggravated brain inflammation caused by pre-existing intra-amniotic inflammation, while antenatal glucocorticoids before LPS administration reduced brain damage [17].

The intricate relationship between chorioamnionitis, perinatal infection, and the use of postnatal corticosteroids in shaping the immature brain has been scarcely explored. This uncertainty raises concerns about the justification for the AAP's recommendation of prophylactic HC in the context of chorioamnionitis. We hypothesized that LPS might sensitize the brain to steroid-induced injury. Therefore, our study aimed to investigate the effects of postnatal Dex or HC therapy on various brain areas following exposure to LPS.

## 2. Materials and Methods

### 2.1. Animals

All experiments were conducted in accordance with the National Institutes of Health Guidelines for Animal Research (Guide for the Care and Use of Laboratory Animals) and approved by the National Cheng Kung University Institutional Animal Care and Use Committee (approval reference number: 111112). All experimental procedures were performed during the light cycle. The pregnant Wistar rats were obtained from the BioLASCO Experimental Animal Center (Taipei, Taiwan) 1 week before giving birth to rat pups. They were housed under a 12-h light/12-h dark cycle (lights on at 7 AM) in a temperature (24°C)- and humidity-controlled room managed by qualified caretakers in the university animal center.

We initially conducted tests to determine the optimal dosage, aiming to replicate the clinical conditions of lung inflammation. Animal models of neonatal lung injury and BPD with a specific focus on postnatal inflammation have been explored [18–21]. We administered intraperitoneal (i.p.) injections of LPS (Sigma Aldrich, LPSs from Escherichia) to neonatal pups within the first 24 h after birth, which was defined as postnatal Day 1 (P1). The doses administered were 0.25 mg/kg, 0.5 mg/kg, 1 mg/kg, and normal saline (Sal), respectively. The Sal group was used as the control. These pups were subsequently sacrificed at P5 to study the induced lung inflammation. We found that both LPS 0.5 or 1 mg/kg resulted in large and simple distal air spaces and increased neutrophil infiltration at P5 (Supporting Information 1: Figure S1). We, therefore, chose LPS 0.5 mg/kg for subsequent experiments to simulate the clinical scenario of extremely preterm infants exposed to perinatal infection.

Rat pups randomly received either a single dose of LPS (i.p., 0.5 mg/kg) or an equivalent volume of normal Sal at P1. Postnatal corticosteroids Dex (Standard Chem & Pharm Co., Ltd, Taiwan) or HC (Pfizer, Puurs, Belgium) with equivalent glucocorticoid potency were given i.p. from P2 to P4 with tapering dosage (Dex: 0.20, 0.10, and 0.05 mg/kg; HC: 5.00, 2.50, and 1.25 mg/kg). The dosage of Dex was modified from those of our previous study (Dex: 0.2 mg/kg from P1 to P3) [22], which used the lowest equivalent dose of Dex to facilitate extubation of mechanically-ventilated infants [23–25]. We found in our previous study that even with such a low dose of Dex, brain weights at P4 were still significantly lower compared with control (unpublished data). We, therefore, used a tapering protocol in the current study.

We employed a two-factor factorial design. The first factor had two levels: LPS-treated and LPS-untreated (sal) groups. The second factor included three levels: Dex, HC, and Sal. This arrangement yielded six groups in total: Sal-Sal, Sal-HC, Sal-Dex, LPS-Sal, LPS-HC, and LPS-Dex. The sample size and sex distribution are as follows: Sal-Sal (8; male: 4, female: 4), Sal-HC (8; male: 2, female: 6), Sal-Dex (8; male: 5, female: 3), LPS-Sal (8; male: 4, female: 4), LPS-HC (8; male: 4, female: 4), and LPS-Dex (8; male: 4, female: 4). These pups were from five litters. Body weights were recorded from P1 to P5, and brain weights were measured at P5. The timing of LPS (P1) and subsequent corticosteroid injection (Dex or HC) (P2–P4) was chosen to mimic perinatal infection and postnatal corticosteroid treatment at a developmental stage of the brain corresponding to human preterm birth (23–32 weeks gestational age).

### 2.2. Brain Specimen Preparation

One day after the last corticosteroid administration (P5), the anesthetized (Zoletil, 0.1 mL/10 g, i.p.) rats were perfused from the left cardiac ventricle with chilled phosphate-buffered saline, then their brains were quickly removed. The left hemispheres were post-fixed with buffered formaldehyde (4% paraformaldehyde solubilized in phosphate-buffered sal, pH 7.4) at 4°C for 2 days, then the paraffin-embedded tissues were sliced into 10-μm thickness sagittal sections using a microtome and mounted onto slides. Slides were then deparaffinized and stained with TUNEL. The right hemispheres were quickly dissected out, frozen in liquid nitrogen, and homogenized with ice-cold commercial tissue protein extraction reagent (78510, Thermo Fisher Scientific Inc., Waltham, MA) containing protease and phosphatase inhibitors (04693116001 & PHOSS-RO, Roche Diagnostics, Mannheim, Germany). The homogenates were centrifuged at 10,000 *g* for 15 min at 4°C. The protein concentrations of the supernatants were determined and adjusted to the same concentration and stored at −80°C until use.

### 2.3. TUNEL Assay

Apoptotic cells of brain sections (1.3 mm lateral to bregma) were stained using Apoptag Plus Fluorescein in a Situ Apoptosis Detection kit (S7111, Merck KGaA, Darmstadt, Germany) before counterstained with mounting medium containing DAPI. Photomicrographs were taken by a digital camera connected to a computer equipped with the TissueFAXS system. The regions of interest were captured by TissueFAXS software and defined by the following criteria: (1) a rat brain atlas, (2) density of DAPI signals, and (3) size of the brain region. To minimize the batch-to-batch variations, each set of staining experiments was performed at the same time. The immunoreactive signals of TUNEL were identified by measuring the signal intensity higher than the background threshold using the ImageJ Fiji software. Furthermore, the background intensity was fixed and applied to all sections.

### 2.4. Determination of Cell Types of TUNEL-Positive Cells

After TUNEL stain, brain sections blocked with 3% normal goat serum (Cat# S26-M, Sigma-Aldrich, St. Louis, MO) were prepared in phosphate-buffered sal with Tween 20 (PBST) for 1 h at room temperature and probed with one of the following primary antibodies: mouse antineuronal nuclear antigen (NeuN) (1:250 dilution, Cat. # MAB377, Merck KGaA), rabbit antiglial fibrillary acidic protein (GFAP) (1:250 dilution, Cat. # Z0334, Dako-Agilent, Santa Clara, CA), rabbit anti-ionized calcium-binding adapter molecule-1 (Iba1) (1:250, Cat. #: 019-19741, Wako Pure Chemical Industries, Osaka, Japan), and rabbit anti-SRY-box transcription factor 10 (SOX10) (1:250 dilution, Cat. #: TA381887, Ori-Gene, Rockville, MA) for 16 h at room temperature. The brain sections were washed and incubated with secondary antibodies for 2 h at room temperature. The antibodies used were as follows: for NeuN, a 1:250 dilution of Alexa 594-conjugated goat anti-mouse IgG (Cat. #A11005, Thermo Fisher Scientific); for GFAP, Iba1, and SOX10, a 1:250 dilution of Alexa 594-conjugated goat anti-rabbit IgG (Cat. #A11012, Thermo Fisher Scientific). Following incubation, the sections were washed with PBST and mounted with DAPI. The immunofluorescent images were captured by an optical fluorescence microscope (Model: Axio Imager A1, Carl Zeiss) equipped with a digital camera (Model: Axiocam 305 Color, Carl Zeiss). ImageJ Fiji software was used to merge the multiple color channels and analyze the parameters of interest.

### 2.5. Western Blot

The relative levels of postsynaptic density protein 95 (PSD95) and synaptophysin in the hippocampus, cortex, thalamus, striatum, brain stem, and cerebellum were determined using western blot. Brain supernatants (10 or 20 µg of protein each) were mixed with a sample buffer containing 2% of 2-mercaptoethanol, heated to 95°C for 10 min to denature the protein, loaded onto polyacrylamide gel (10%), and resolved at 120 V for 2 h. The separated proteins were transferred to a polyvinylidene fluoride membrane (IPVH00010, Merck KGaA) blocked with 5% skimmed milk and hybridized with primary antibodies overnight at 4°C as follows: mouse anti-PSD95 (1:1000, Cat. #: 610495, BD Biosciences, Franklin Lakes, NJ) and mouse anti-synaptophysin (1:1000, Cat. #: MAB5258-1, Merck KGaA). The β-actin (1:10,000. Cat. #: MAB1501R, Merck KGaA) was used as a loading control. After washing, the membranes were hybridized with proper horseradish peroxidase-conjugated secondary antibodies (Jackson ImmunoResearch Inc., West Grove, PA). The bound antibodies were detected using an enhanced chemiluminescence detection kit (WBKLS0500, Merck KGaA) and x-ray film. Relative protein expressions were estimated by normalizing with levels of β-actin. The band densities were analyzed using ImageJ Fiji software (version 1.51 K). For reprobing, the bound antibodies were removed from the membranes by incubating the membranes with stripping buffer containing 2% of SDS, 62.5 mM of tris, and 0.8% of 2-mercaptoethanol for 20 min at 55°C. The conditions of the dilution ratios of antibodies and the image exposure time were tested to confirm that the luminescence signals were within the linear range of detection.

### 2.6. Reverse Transcription-Quantitative Polymerase Chain Reaction (RT-qPCR)

RNA from all brain regions was extracted by using Quick-RNA Miniprep Plus Kit (Cat. #: R1058, Zymo Research, Irvine, CA). After extraction, RNAs (100 ng) were transcripted into cDNA by using the PrimeScriptTM RT reagent Kit (Cat. #RR037A, Takara Bio, Shiga, Japan). Real-time PCR was performed using Fast SYBR Green Master Mix, (Cat. # 4385616, Thermo Fisher Scientific) and a final concentration of 10 μM gene specific primer. All qPCR analyses were conducted utilizing the StepOnePlus System (Cat. # 4376357, Thermo Fisher Scientific). Primers for mRNA expression are shown in Supporting Information 2: Table S1. PCR signals were compared among groups after normalization using GAPDH RNA expression as the internal reference, calculated using the inverse log of ΔΔCT.

### 2.7. Statistical Analysis

Quantitative data are expressed as mean ± standard deviation (SD). Significance was set at *p* < 0.05. Two-way ANOVA was used to analyze comparisons involving two independent variables (e.g., LPS and steroids). If significant main effects or interactions were identified, post hoc analyses were conducted using Bonferroni's multiple comparisons test. Exact *p*-values for all comparisons are provided in Supporting Information 3: Table S2.

## 3. Results

### 3.1. Effect of LPS and Postnatal Corticosteroid on Body Weight and Brain Weight

**Figure 1:**
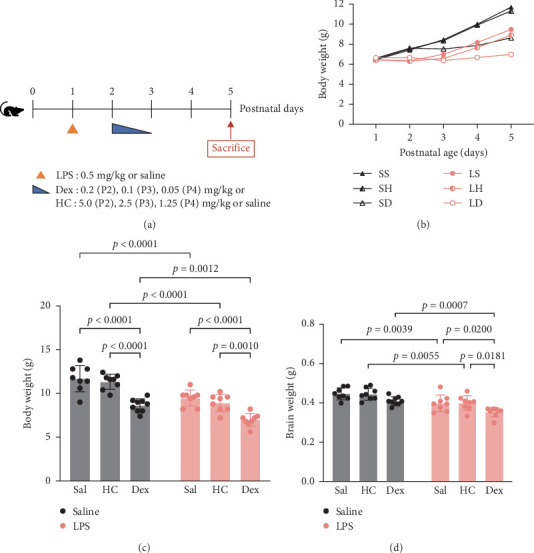
The effects of postnatal LPS administration followed by corticosteroid treatment on body and brain weight gains. (A) The experimental protocol. (B) Mean body weights of rat pups from postnatal Day 1 (P1) to P5. SS, Sal–Sal; SH, Sal-HC; SD, Sal-Dex; LS, LPS-Sal; LH, LPS-HC; LD, LPS-Dex. (C) Body weights at P5. (D) Brain weights at P5. *n* = 8 in each group. Data are expressed as mean ± SD. The details of statistical analysis are described in Supporting Information 3: Table S2.

We examined how LPS and/or postnatal corticosteroid affects body weight and brain growth. The experimental protocol is illustrated in Figure 1A. Among rats of the LPS groups, after the administration of LPS at P1, weight growth stopped for 1 day and started to catch up since P3 in LPS-Sal and LPS-HC groups, while growth retardation persisted till the end of the experiment on P5 in LPS-Dex group. In the Sal control groups, the weight growth remained comparable between the Sal-Sal and Sal-HC groups, while growth fell behind throughout the experiment in the Sal-Dex group (Figure 1B). Two-way ANOVA revealed that both corticosteroids (*p* < 0.0001) and LPS (*p* < 0.0001) have significant effects on body weight (Figure 1C). Post hoc analyses indicated that there were no significant differences in weight gain between the HC and Sal groups; however, Dex had a significant effect on growth. Furthermore, there was no interaction between LPS and corticosteroid (*p*  > 0.5), suggesting that the influence of a single dose of LPS on growth was transient.

In Sal control groups, neither HC nor Dex had any impact on brain weight (Figure 1D). However, LPS significantly reduced brain weight (Figure 1D, *p* < 0.001) in the LPS groups. Post hoc analyses revealed that all three LPS groups had smaller brain weights than their respective Sal control groups (Figure 1D). Moreover, Dex further aggravated this effect. The brain weights of the LPS-Dex group showed significant decreases compared to both the LPS-Sal group (*p* < 0.05) and the LPS-HC group (*p* < 0.05).

### 3.2. LPS Followed by Early Postnatal Corticosteroid Exposure Induced Cell Death to Possible Apoptosis in the Cerebellum and Brain Stem

**Figure 2:**
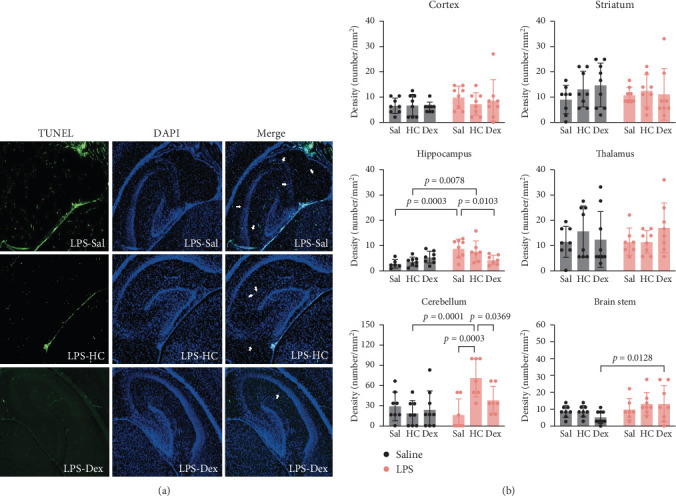
Immunofluorescence analysis of TUNEL-positive cells in various brain regions. (A) Representative immunomicrographs in the hippocampus. White arrows indicate apoptotic cells. (B) Quantitative results of TUNEL stain. Data are expressed as mean ± SD. *n* = 8 in each group. The details of statistical analysis are described in Supporting Information 3: Table 2.

We investigated whether LPS and/or postnatal corticosteroid treatment induces cell apoptosis in six brain regions (cortex, striatum, hippocampus, thalamus, cerebellum, and brain stem) of rat pups. The extent of cell death to possible apoptosis, indicated by TUNEL-positive cells, is shown in Figure 2A. In general, the numbers of apoptotic cells in all selected brain regions were low on P5. Among all brain regions, the cerebellum had the highest number of TUNEL-positive cells. Two-way ANOVA revealed a significant interaction between LPS and corticosteroid in the cerebellum. Post hoc analyses indicated that the LPS-HC group had significantly higher numbers of apoptotic cells than the Sal-HC group (*p* < 0.001; Figure 2B), indicating a possible LPS-sensitizing effect. No significant differences were found between the LPS-Dex and Sal-Dex groups. A similar LPS sensitizing effect could be observed in the brain stem region, with the LPS-Dex group showing significantly higher numbers of apoptotic cells compared with the Sal-Dex group (*p* < 0.05). We found that LPS also increased the density of TUNEL-positive cells in the hippocampus; However, the addition of HC or Dex paradoxically decreased the number of TUNEL-positive cells in this region (Figure 2B).

**Figure 3:**
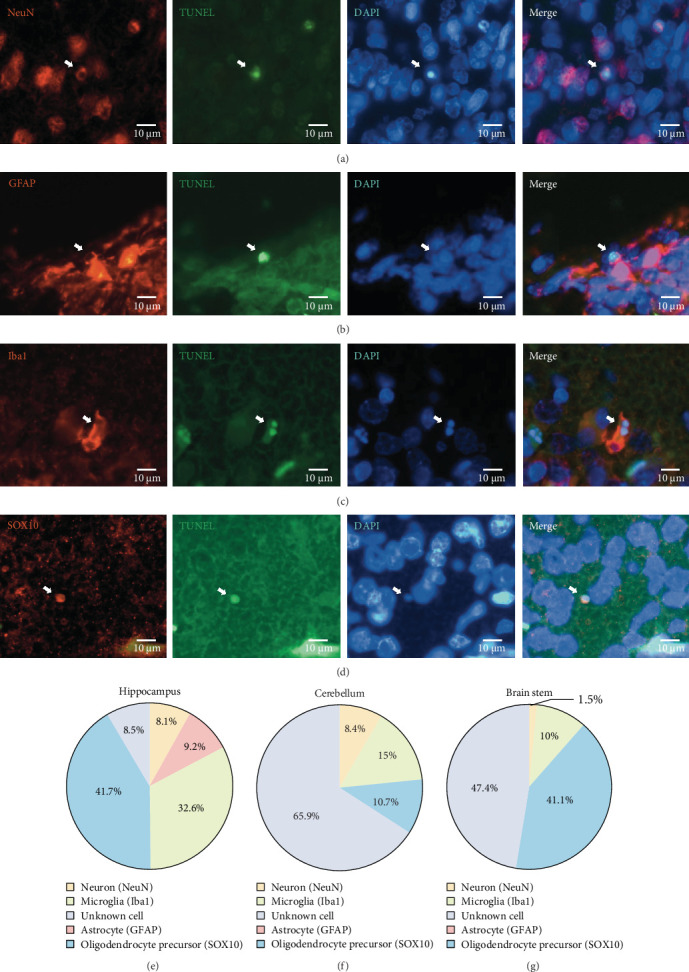
Determination of TUNEL-positive cells. Sections from three brain regions (hippocampus, cerebellum, and brain stem) were stained using NeuN, GFAP, Iba1, and SOX10 to identify neurons, astrocytes, microglia, and oligodendrocyte precursor cells, respectively in TUNEL^+^ cells. (A–D) Representative immunofluorescent micrographs of dual NeuN^+^/TUNEL^+^ (A), GFAP^+^/TUNEL^+^ (B), Iba1^+^/TUNEL^+^ (C), and SOX10^+^/TUNEL^+^ (D) cells. White arrows indicate dual positive stained cells. DAPI staining was used to confirm the presence of cell nuclei. (E–G) Quantitative results on TUNEL-positive cell types presented in pie charts for three different brain regions.

To determine the cell type of TUNEL-positive cells, we performed staining on three brain regions (i.e., hippocampus, cerebellum, and brain stem), where significant increase in TUNEL-positive cells was observed following LPS and postnatal corticosteroids treatment. The sections were stained using NeuN, GFAP, Iba1, and SOX10 to identify neurons, astrocytes, microglia, and oligodendrocyte precursor cells, respectively (Figure 3). The results showed that, on average, most TUNEL-positive cells in the hippocampus and brain stem were oligodendrocyte precursors, followed by microglia, with neurons and astrocytes being the least common; However, the majority of TUNEL-positive cells did not fall into any of these four cell types in the cerebellum (Figure 3).

### 3.3. LPS Followed by Early Postnatal Corticosteroid Exposure Disrupted Synaptic Integrity in the Hippocampus

We further investigated how LPS and/or postnatal corticosteroid exposure affects synaptic growth in the pup's brain. The synaptic integrity of different brain regions was evaluated by measuring two synaptic marker proteins: synaptophysin (presynaptic) and PSD-95 (postsynaptic).

**Figure 4:**
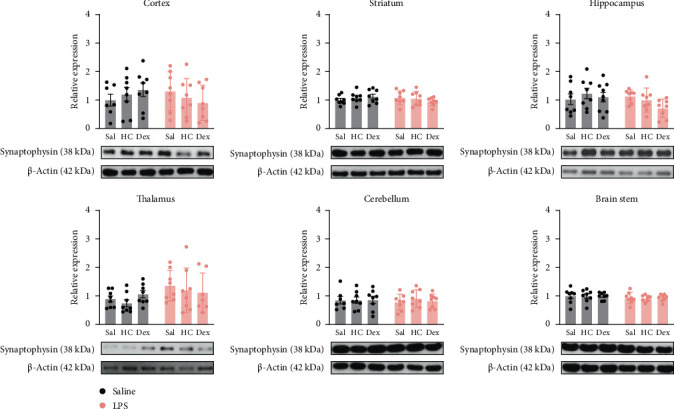
Quantitative results of synaptophysin levels in various brain regions. Data are expressed as mean ± SD. *n* = 8 or 7 after removing an outlier. Please refer to Supporting Information 1: Figure S8 for whole western blots. The details of statistical analysis are described in Supporting Information 3: Table S2.

**Figure 5:**
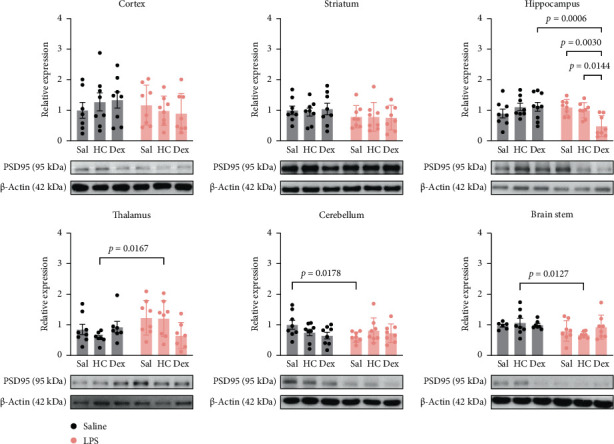
Quantitative results of PSD95 levels in various brain regions. Data are expressed as mean ± SD. *n* = 8 or 7 after removing an outlier. Please refer to Supporting Information 1: Figure S8 for whole western blots. The details of statistical analysis are described in Supporting Information 3: Table S2.

Western blot results showed that synaptophysin levels remained unchanged in all six selected brain regions regardless of LPS administration or exposure to HC or Dex (Figure 4). However, LPS administration followed by Dex significantly reduced PSD95 levels compared with Dex alone (Sal-Dex) in the hippocampus (Figure 5). Furthermore, in the brain stem, LPS followed by HC showed significantly lower PSD95 levels compared to corresponding sal controls. Neither HC nor Dex alone affected PSD95 expression in any brain region in the Sal control groups (Figure 5).

We found no differences in mRNA expression of synaptophysin (Supporting Information 1: Figure S2) and PSD95 (Supporting Information 1: Figure S3) in all six brain regions. Furthermore, there were no sex-specific differences in any of the measured outcomes, including body and brain weight (Supporting Information 1: Figure S4), quantification of TUNEL-positive cells (Supporting Information 1: Figure S5), or protein levels of synaptophysin (Supporting Information 1: Figure S6) and PSD95 (Supporting Information 1: Figure S7). However, the statistical power was insufficient to confirm these results with certainty and requires further validation.

### 3.4. Region-Specific Changes in Glucocorticoid Receptor (GR) and Mineralocorticoid Receptor (MR) mRNA Expression After LPS and Corticosteroid Treatment

**Figure 6:**
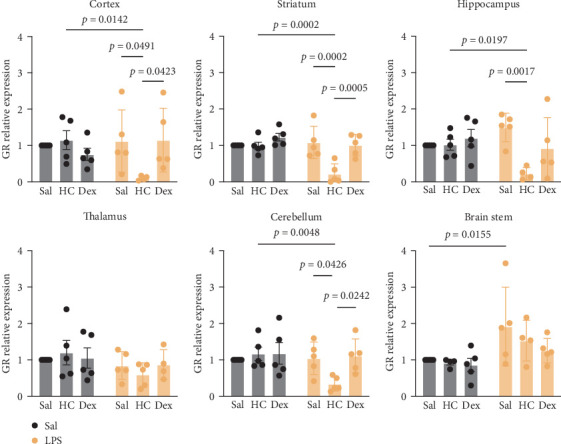
Quantitative results of glucocorticoid receptor (GR) mRNA expression in different brain regions. Data are expressed as mean ± SD. *n* = 5 or 4 after removing an outlier. The details of statistical analysis are described in Supporting Information 3: Table S2.

**Figure 7:**
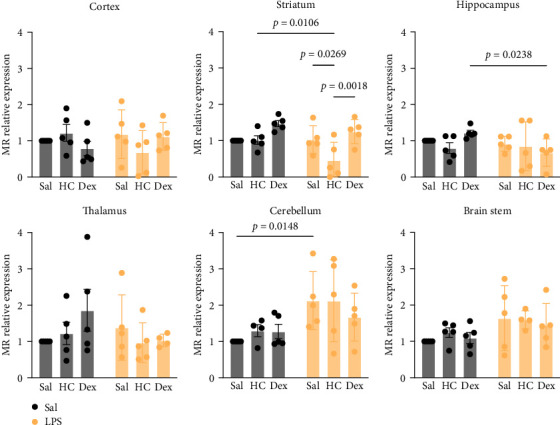
Quantitative results of mineralocorticoid receptor (MR) mRNA expression in different brain regions. Data are expressed as mean ± SD. *n* = 5 or 4 after removing an outlier. The details of statistical analysis are described in Supporting Information 3: Table S2.

GR were detected in all examined brain regions, with no statistically significant differences in expression levels across regions. In contrast, MR expression was generally low in most areas, except for the cortex and hippocampus (Supporting Information 1: Figure S9). Corticosteroid treatment alone (with either HC or Dex) did not significantly alter GR mRNA levels in any brain region. However, administration of LPS followed by HC led to a significant reduction in GR mRNA levels in the cortex, striatum, hippocampus, and cerebellum (Figure 6). A similar reduction in MR mRNA was observed in the striatum, but not in other brain regions (Figure 7).

## 4. Discussion

This study demonstrated in a neonatal rat model that LPS administration on P1 sensitized the immature brain to subsequent corticosteroid treatment, resulting in cell death to possible apoptosis, especially in the cerebellum, and disrupting synaptic integrity in the hippocampus and brain stem on P5. Oligodendrocyte precursor cells represented the major cell type among TUNEL-positive cells in these regions. Without prior LPS exposure, corticosteroid treatment did not affect the density of TUNEL-positive cells nor the expressions of synaptic proteins.

The 2022 American Academy of Pediatrics guideline on the use of postnatal corticosteroids indicated that early (<7 days old) low-dose HC may prevent BPD or death in infants weighing less than 1000 g and exposed to chorioamnionitis [26], based on a meta-analysis showing that chorioamnionitis was an independent predictor of response to HC [27]. However, early HC therapy may be associated with a higher rate of neurologic dysfunction and lower performance IQ in children of preschool age as compared with placebo [28]. Furthermore, the sensitizing effects of the preceding infection/inflammation have rarely been considered. It was shown in human infants that the combination of maternal infection and asphyxia amplifies the risk of cerebral palsy [15, 29]. Administration of LPS to newborn rat pups sensitizes the immature brain to subsequent hypoxic-ischemic injury [30–33]. However, the association between infection, hypoxic-ischemic insult, and cerebral injury is not uniform and is dependent on the maturity of the animal. For example, LPS pretreatment in adult mice may have a protective preconditioning effect upon hypoxic-ischemic insult [34]. It was shown that increasing brain maturity (postnatal age) and the concurrent rise in cerebral expression of toll-like receptor 4 are critical for the protective preconditioning effects of LPS [35].

Only a few studies examined how infection followed by corticosteroid treatment affects the neonatal brain [17]. The timing of LPS exposure and corticosteroid treatment is crucial in determining their impact on the fetal immune response. It has been demonstrated that antenatal glucocorticoid administration before the LPS exposure prevented inflammation in the fetal lung, thymus, and brain; whereas, glucocorticoid administration after the LPS exposure aggravated inflammation in the fetal organs [36, 37]. In line with their findings, we also observed increased neuron cell death and decreased levels of postsynaptic protein PSD95 when corticosteroids were administered after exposure to LPS. Moreover, the interval between LPS and glucocorticoid and how it affects the outcomes of target organs has been rarely investigated; one of the aforementioned studies showed that antenatal LPS followed by glucocorticoid given 7 days later worsened inflammatory changes and apoptosis of the fetal brain, especially in the white matter [36]. Our study focuses on the acute effects of LPS and corticosteroid exposure during the first postnatal days (P1–P5) in rats, a period equivalent to late gestation/early preterm human brain development marked by rapid oligodendrocyte maturation, synaptogenesis, and gliogenesis [38]. This critical window features intense oligodendrocyte precursor cells proliferation and synaptic formation, processes highly sensitive to perinatal insults that may cause lasting brain injury [39]. Our analysis at P5 captured immediate cellular and molecular responses to combined inflammatory and steroid insults during this vulnerable phase, revealing damages to oligodendrocyte precursor cells and synaptic integrity. However, further investigation into the long-term persistence and functional consequences of these early changes requires extended longitudinal studies.

Another important finding of the current study is the vulnerability of the LPS-sensitized cerebellum to corticosteroid treatment. Clinical studies and animal models provide evidence that exposure to systemic inflammation in the fetal or neonatal periods is associated with cerebellar maldevelopment [40–42]. Yet, there is a lack of human studies examining the effects of glucocorticoids on the cerebellum. A study in rodent showed that there exists a window of vulnerability (between P4 and P10, corresponding approximately to 20 weeks' gestation to 6.5 weeks after birth in humans) during which a single dose of Dex exposure (any dose higher than 0.1 mg/kg) can produce apoptosis of neural progenitor cells in the cerebellar external granule layer and cause permanent reductions in neuronal cell counts [43]. Our study, with a tapering dose of Dex (0.2, 0.1, and 0.05 mg/kg) or HC with equivalent glucocorticoid potency, from P2 to P4, did not show significant cell death to possible apoptosis with either Dex or HC alone. However, when HC was administered after LPS treatment, we observed a striking increase in cell death to possible apoptosis in the cerebellar cortex. Yet, this phenomenon was not obvious in the LPS-Dex group. We observed region-specific changes in GR and MR mRNA expression in the neonatal brain following combined LPS and corticosteroid exposure. In sal-control animals, GRs were broadly expressed across all examined brain regions without significant regional differences, whereas MR expression was generally low in most areas, except notably in the cortex and hippocampus. These patterns align with previous foundational studies on corticosteroid receptor distribution in the brain [44, 45]. After LPS and HC treatment, GR mRNA levels were reduced in the cortex, striatum, hippocampus, and cerebellum, while MR mRNA decrease was primarily observed in the striatum. Earlier research has shown that following LPS exposure, the decrease in steroid hormone receptors may set the stage for reduced anti-inflammatory effects of endogenous steroids on the immune system, leading to prolonged microglial activation [46]. Our findings of selective increased apoptosis in the cerebellum despite decreased GR mRNA across multiple brain regions can be explained by the cerebellum's distinct developmental timeline and sensitivity to injury. The cerebellum undergoes prolonged postnatal maturation marked by extensive proliferation, migration, and differentiation of granule cell precursors, processes that extend well beyond the neonatal period [47, 48]. Previous research showed that neonatal LPS exposure causes long-lasting cellular and molecular changes specifically in the cerebellum, supporting its selective susceptibility [49]. Many studies highlight a unique profile of cerebellar microglial activation in response to inflammatory stimuli, including elevated proinflammatory cytokines and morphological changes distinct from other brain regions [50]. These studies suggest that cerebellar microglia may play a role in region-specific neuroinflammation and vulnerability, which is consistent with our observed selective increase in cell death, possibly due to apoptosis, in the cerebellum following combined LPS and HC exposure. In contrast, other brain regions, such as the cortex and striatum mature earlier and exhibit greater resistance to apoptosis despite similar molecular disruptions [51]. On the other hand, our results also suggested that Dex's effect on hippocampal synaptic proteins PSD95 appeared to depend on neuroinflammatory priming. While GR mRNA is stable and MR expression is relatively high in the hippocampus—where MR may help maintain synaptic stability under basal (unstressed) conditions [52]—LPS-induced inflammation likely sensitizes neurons to Dex's GR-mediated effects. Transcriptomic and electrophysiological studies showed that Dex alone minimally affects synaptic plasticity-related genes and proteins but significantly alters synaptic remodeling and neurotransmission pathways under inflammatory conditions, disrupting the balance of MR and GR signaling [53, 54]. These findings highlight the receptor- and context-dependent nature of Dex's modulation of hippocampal synaptic architecture, where inflammation enhances its impact on synaptic proteins and plasticity, rather than through direct anti-inflammatory action alone. Future work measuring serum steroid levels, receptor occupancy, the enzymatic activity of 11 β-hydroxysteroid dehydrogenase type 1 and 2, and the interaction with other transcriptional co-regulators could further clarify these mechanisms [55–57].

We found that LPS and subsequent glucocorticoid treatment increased cell death to possible apoptosis of oligodendrocyte precursor cells, especially in the hippocampus. Previous studies have shown the detrimental effects of LPS on oligodendrocyte differentiation [58–60]. One study reported that exposure to in-utero glucocorticoid disrupted fetal astrocyte development [61]. However, the dual-hit effect of LPS and glucocorticoid on glial cells remained to be elucidated. Nevertheless, the key role of glial cell dysfunction (including microgliosis, astrogliosis, and oligodendrocyte injury) in encephalopathy of prematurity has been established [62]. Our findings, although still preliminary, contribute to this growing field of interest.

Our study has several limitations. The mechanisms of how LPS sensitizes the immature brain to cell death to possible apoptosis and synaptic loss remain unknown. The doses of LPS or corticosteroids and the time window of vulnerability are still unclear. We administered minimal doses of LPS and corticosteroids to minimize their individual effects and to examine their combined adverse outcomes. We are still uncertain about the impact of brain injuries during the neonatal period on future cognitive and behavioral changes.

In conclusion, we found that LPS sensitizes the immature brain to subsequent glucocorticoid treatment, exacerbating cell death to possible apoptosis and synaptic loss in some brain regions. These findings highlight the complex interaction between infection and corticosteroid therapy in neonatal brain development.

## Data Availability

The data that support the findings of this study are available from the corresponding author upon reasonable request.

## Supporting Information

Supporting Information 1 Figure S1. The effect of single postnatal LPS administration on the rat pup lung. Representative H&E images of rat lungs harvested at postnatal Day 5 (P5). *n* = 3 in each group. Figure S2. Quantitative results of synaptophysin mRNA levels in various brain regions. Data are expressed as mean ± SD. *n* = 6; in certain cases, it is reduced to *n* = 5 due to the exclusion of outliers. Two-way ANOVA followed by Bonferroni's multiple comparisons was used to analyze differences among treatment (Sal and LPS) and drug (Sal, HC, and Dex). The details of statistical analysis are described in Supporting Information 3: Table S2. In the cerebellum, synaptophysin mRNA level was significantly higher in LPS-Sal group compared with Sal-Sal group. Figure S3. Quantitative results of PSD95 mRNA levels in various brain regions. Data are expressed as mean ± SD. *n* = 6; in certain cases, it is reduced to *n* = 5 due to the exclusion of outliers. Two-way ANOVA followed by Bonferroni's multiple comparisons was used to analyze differences among treatment (Sal and LPS) and drug (Sal, HC, and Dex). The details of statistical analysis are described in Supporting Information 3: Table S2. Figure S4. Body weight and brain weight by LPS vs. normal saline control and by sex. Data were expressed as mean ± SD. Three-way ANOVA followed by Bonferroni's multiple comparisons was used to analyze differences among treatment (Sal and LPS), drug (Sal, HC, and Dex) and sex (male and female). Sal-Sal (*n* = 8; male: 4, female: 4), Sal-HC (*n* = 8; male: 2, female: 6), Sal-Dex (*n* = 8; male: 5, female: 3), LPS-Sal (*n* = 8; male: 4, female: 4), LPS-HC (*n* = 8; male: 4, female: 4), and LPS-Dex (*n* = 8; male: 4, female: 4). The details of statistical analysis are described in Supporting Information 3: Table S2. Figure S5. Quantification of TUNEL stain in different brain regions, by LPS vs. normal saline control and by sex. Data were expressed as mean ± SD. Three-way ANOVA followed by Bonferroni's multiple comparisons was used to analyze differences among treatment (Sal and LPS), drug (Sal, HC, and Dex) and sex (male and female). Sal-Sal (*n* = 8; male: 4, female: 4), Sal-HC (*n* = 8; male: 2, female: 6), Sal-Dex (*n* = 8; male: 5, female: 3), LPS-Sal (*n* = 8; male: 4, female: 4), LPS-HC (*n* = 8; male: 4, female: 4), and LPS-Dex (*n* = 8; male: 4, female: 4). The details of statistical analysis are described in Supporting Information 3: Table S2. Figure S6. Quantification of synaptophysin protein level (relative to control) in different brain regions, by LPS vs. normal saline control and by sex. Data were expressed as mean ± SD. Three-way ANOVA followed by Bonferroni's multiple comparisons was used to analyze differences among treatment (Sal and LPS), drug (Sal, HC, and Dex) and sex (male and female). Sal-Sal (*n* = 8; male: 4, female: 4), Sal-HC (*n* = 8; male: 2, female: 6), Sal-Dex (*n* = 8; male: 5, female: 3), LPS-Sal (*n* = 8; male: 4, female: 4), LPS-HC (*n* = 8; male: 4, female: 4), and LPS-Dex (*n* = 8; male: 4, female: 4). The details of statistical analysis are described in Supporting Information 3: Table S2. Figure S7. Quantification of PSD95 protein level (relative to control) in different brain regions, by LPS vs. normal saline control and by sex. Data were expressed as mean ± SD. Three-way ANOVA followed by Bonferroni's multiple comparisons was used to analyze differences among treatment (Sal and LPS), drug (Sal, HC, and Dex) and sex (male and female). Sal-Sal (*n* = 8; male: 4, female: 4), Sal-HC (*n* = 8; male: 2, female: 6), Sal-Dex (*n* = 8; male: 5, female: 3), LPS-Sal (*n* = 8; male: 4, female: 4), LPS-HC (*n* = 8; male: 4, female: 4), and LPS-Dex (*n* = 8; male: 4, female: 4). The details of statistical analysis are described in Supporting Information 3: Table S2. Figure S8. Whole gel images of western blots. This image shows the unedited western blot images of Synaptophysin, PSD95, and β-actin across all six brain regions. SS: Sal-Sal; SH: Sal-HC; SD: Sal-Dex; LS: LPS-Sal; LH: LPS-HC; LD: LPS-Dex. Figure S9. Comparative results of glucocorticoid receptor (GR) and mineralocorticoid (MR) mRNA levels in various brain regions in saline control animals. Data are expressed as mean ± SD. *n* = 5; in certain cases, it is reduced to *n* = 4 due to the exclusion of outliers.

Supporting Information 2 Table S1. Primer sequences for RT-qPCR.

Supporting Information 3 Table S2. Statistical analysis and results.

## References

[1] Nakashima T., Inoue H., Sakemi Y., Ochiai M., Yamashita H., Ohga S. (2021). Trends in Bronchopulmonary Dysplasia Among Extremely Preterm Infants in Japan, 2003–2016. *The Journal of Pediatrics*.

[2] Bancalari E., Jain D. (2019). Bronchopulmonary Dysplasia: 50 Years After the Original Description. *Neonatology*.

[3] Salimi U., Dummula K., Tucker M. H., Dela Cruz C. S., Sampath V. (2022). Postnatal Sepsis and Bronchopulmonary Dysplasia in Premature Infants: Mechanistic Insights Into “New BPD“. *American Journal of Respiratory Cell and Molecular Biology*.

[4] Sweet D. G., Carnielli V. P., Greisen G. (2023). European Consensus Guidelines on the Management of Respiratory Distress Syndrome: 2022 Update. *Neonatology*.

[5] Doyle L. W., Cheong J. L., Ehrenkranz R. A., Halliday H. L. (2017). Early (<8 Days) Systemic Postnatal Corticosteroids for Prevention of Bronchopulmonary Dysplasia in Preterm Infants. *The Cochrane Database of Systematic Reviews*.

[6] Doyle L. W., Cheong J. L., Ehrenkranz R. A., Halliday H. L. (2017). Late (>7 Days) Systemic Postnatal Corticosteroids for Prevention of Bronchopulmonary Dysplasia in Preterm Infants. *The Cochrane Database of Systematic Reviews*.

[7] Huang C.-C., Lin H.-R., Liang Y.-C., Hsu K.-S. (2007). Effects of Neonatal Corticosteroid Treatment on Hippocampal Synaptic Function. *Pediatric Research*.

[8] Feng Y., Kumar P., Wang J., Bhatt A. J. (2015). Dexamethasone But Not the Equivalent Doses of Hydrocortisone Induces Neurotoxicity in Neonatal Rat Brain. *Pediatric Research*.

[9] Trousson C., Toumazi A., Bourmaud A., Biran V., Baud O. (2023). Neurocognitive Outcomes at Age 5 years After Prophylactic Hydrocortisone in Infants Born Extremely Preterm. *Developmental Medicine & Child Neurology*.

[10] Baud O., Trousson C., Biran V., Leroy E., Mohamed D., Alberti C. (2017). Association Between Early Low-Dose Hydrocortisone Therapy in Extremely Preterm Neonates and Neurodevelopmental Outcomes at 2 Years of Age. *JAMA*.

[11] Alison M., Tilea B., Toumazi A. (2020). Prophylactic Hydrocortisone in Extremely Preterm Infants and Brain MRI Abnormality. *Archives of Disease in Childhood - Fetal and Neonatal Edition*.

[12] Dubner S. E., Rickerich L., Bruckert L. (2024). Early, Low-Dose Hydrocortisone and Near-Term Brain Connectivity in Extremely Preterm Infants. *Pediatric Research*.

[13] Garnier Y., Berger R., Alm S. (2006). Systemic Endotoxin Administration Results in Increased S100B Protein Blood Levels and Periventricular Brain White Matter Injury in the Preterm Fetal Sheep. *European Journal of Obstetrics & Gynecology and Reproductive Biology*.

[14] Duncan J. R., Cock M. L., Scheerlinck J.-P. Y. (2002). White Matter Injury After Repeated Endotoxin Exposure in the Preterm Ovine Fetus. *Pediatric Research*.

[15] Wu Y. W., Escobar G. J., Grether J. K., Croen L. A., Greene J. D., Newman T. B. (2003). Chorioamnionitis and Cerebral Palsy in Term and Near-Term Infants. *JAMA*.

[16] Stone A. C., Strickland K. C., Tanaka D. T., Gilner J. B., Lemmon M. E., Russ J. B. (2023). The Association of Placental Pathology and Neurodevelopmental Outcomes in Patients With Neonatal Encephalopathy. *Pediatric Research*.

[17] Kuypers E., Jellema R. K., Ophelders D. R. (2013). Effects of Intra-Amniotic Lipopolysaccharide and Maternal Betamethasone on Brain Inflammation in Fetal Sheep. *PloS ONE*.

[18] Choi C. W., Kim B. I., Hong J.-S., Kim E.-K., Kim H.-S., Choi J.-H. (2009). Bronchopulmonary Dysplasia in a Rat Model Induced by Intra-Amniotic Inflammation and Postnatal Hyperoxia: Morphometric Aspects. *Pediatric Research*.

[19] Bhandari V. (2014). Postnatal Inflammation in the Pathogenesis of Bronchopulmonary Dysplasia. *Birth Defects Research Part A: Clinical and Molecular Teratology*.

[20] Choi C. W., Lee J., Oh J. Y., Lee S. H., Lee H. J., Kim B. I. (2016). Protective Effect of Chorioamnionitis on the Development of Bronchopulmonary Dysplasia Triggered by Postnatal Systemic Inflammation in Neonatal Rats. *Pediatric Research*.

[21] Lee H. J., Kim B. I., Choi E. S. (2012). Effects of Postnatal Dexamethasone or Hydrocortisone in a Rat Model of Antenatal Lipopolysaccharide and Neonatal Hyperoxia Exposure. *Journal of Korean Medical Science*.

[22] Chang Y.-S., Hou S.-Y., Yu S.-S. (2022). Postnatal Dexamethasone Therapy Impairs Brown Adipose Tissue Thermogenesis and Autophagy Flux in Neonatal Rat Pups. *Theranostics*.

[23] Yates H. L., Newell S. J. (2011). Minidex: Very Low Dose Dexamethasone (0.05 mg/kg/day) in Chronic Lung Disease. *Archives of Disease in Childhood - Fetal and Neonatal Edition*.

[24] Nair A. B., Jacob S. (2016). A Simple Practice Guide for Dose Conversion Between Animals and Human. *Journal of Basic and Clinical Pharmacy*.

[25] Tanney K., Davis J., Halliday H. L., Sweet D. G. (2011). Extremely Low-Dose Dexamethasone to Facilitate Extubation in Mechanically Ventilated Preterm Babies. *Neonatology*.

[26] Cummings J. J., Pramanik A. K., Committee on Fetus and Newborn (2022). Postnatal Corticosteroids to Prevent or Treat Chronic Lung Disease Following Preterm Birth. *Pediatrics*.

[27] Zhou J., Yu Z., Chen C. (2021). Hydrocortisone for Preventing Mortality and Bronchopulmonary Dysplasia in Preterm Infants With or Without Chorioamnionitis Exposure: A Meta-Analysis of Randomized Trials. *American Journal of Perinatology*.

[28] Peltoniemi O. M., Lano A., Yliherva A., Kari M. A., Hallman M. (2016). Randomised Trial of Early Neonatal Hydrocortisone Demonstrates Potential Undesired Effects on Neurodevelopment at Preschool Age. *Acta Paediatrica*.

[29] Onland W., Cools F., Kroon A. (2019). Effect of Hydrocortisone Therapy Initiated 7 to 14 Days After Birth on Mortality or Bronchopulmonary Dysplasia Among Very Preterm Infants Receiving Mechanical Ventilation: A Randomized Clinical Trial. *JAMA*.

[30] Larouche A., Roy M., Kadhim H., Tsanaclis A. M., Fortin D., Sébire G. (2005). Neuronal Injuries Induced by Perinatal Hypoxic-Ischemic Insults Are Potentiated by Prenatal Exposure to Lipopolysaccharide: Animal Model for Perinatally Acquired Encephalopathy. *Developmental Neuroscience*.

[31] Coumans A. B. C., Middelanis J. S., Garnier Y. (2003). Intracisternal Application of Endotoxin Enhances the Susceptibility to Subsequent Hypoxic-Ischemic Brain Damage in Neonatal Rats. *Pediatric Research*.

[32] Martinello K. A., Meehan C., Avdic-Belltheus A. (2019). Acute LPS Sensitization and Continuous Infusion Exacerbates Hypoxic Brain Injury in a Piglet Model of Neonatal Encephalopathy. *Scientific Reports*.

[33] Eklind S., Mallard C., Leverin A. L. (2001). Bacterial Endotoxin Sensitizes the Immature Brain to Hypoxic–Ischaemic Injury. *The European Journal of Neuroscience*.

[34] Wang X., Hagberg H., Nie C., Zhu C., Ikeda T., Mallard C. (2007). Dual Role of Intrauterine Immune Challenge on Neonatal and Adult Brain Vulnerability to Hypoxia-Ischemia. *Journal of Neuropathology and Experimental Neurology*.

[35] Hickey E., Shi H., Van Arsdell G., Askalan R. (2011). Lipopolysaccharide-Induced Preconditioning Against Ischemic Injury Is Associated With Changes in Toll-Like Receptor 4 Expression in the Rat Developing Brain. *Pediatric Research*.

[36] Kuypers E., Collins J. J. P., Jellema R. K. (2012). Ovine Fetal Thymus Response to Lipopolysaccharide-Induced Chorioamnionitis and Antenatal Corticosteroids. *PloS ONE*.

[37] Kuypers E., Collins J. J. P., Kramer B. W. (2012). Intra-Amniotic LPS and Antenatal Betamethasone: Inflammation and Maturation in Preterm Lamb Lungs. *American Journal of Physiology - Lung Cellular and Molecular Physiology*.

[38] Semple B. D., Blomgren K., Gimlin K., Ferriero D. M., Noble-Haeusslein L. J. (2013). Brain Development in Rodents and Humans: Identifying Benchmarks of Maturation and Vulnerability to Injury Across Species. *Progress in Neurobiology*.

[39] Kinney H. C., Volpe J. J. (2018). Myelination Events, Patterns, and Mechanisms in the Human Fetal Brain. *Volpe’s Neurology of the Newborn*.

[40] Bartha A. I., Foster-Barber A., Miller S. P. (2004). Neonatal Encephalopathy: Association of Cytokines With MR Spectroscopy and Outcome. *Pediatric Research*.

[41] Ranger M., Zwicker J. G., Chau C. M. Y. (2015). Neonatal Pain and Infection Relate to Smaller Cerebellum in Very Preterm Children at School Age. *The Journal of Pediatrics*.

[42] Hutton L. C., Yan E., Yawno T., Castillo-Melendez M., Hirst J. J., Walker D. W. (2014). Injury of the Developing Cerebellum: A Brief Review of the Effects of Endotoxin and Asphyxial Challenges in the Late Gestation Sheep Fetus. *The Cerebellum*.

[43] Noguchi K. K., Walls K. C., Wozniak D. F., Olney J. W., Roth K. A., Farber N. B. (2008). Acute Neonatal Glucocorticoid Exposure Produces Selective and Rapid Cerebellar Neural Progenitor Cell Apoptotic Death. *Cell Death & Differentiation*.

[44] Reul J. M. H. M., de Kloet E. R. (1985). Two Receptor Systems for Corticosterone in Rat Brain: Microdistribution and Differential Occupation. *Endocrinology*.

[45] Joëls M., de Kloet E. R. (1994). Mineralocorticoid and Glucocorticoid Receptors in the Brain. Implications for Ion Permeability and Transmitter Systems. *Progress in Neurobiology*.

[46] Sierra A., Gottfried-Blackmore A., Milner T. A., McEwen B. S., Bulloch K. (2008). Steroid Hormone Receptor Expression and Function in Microglia. *Glia*.

[47] Limperopoulos C., Soul J. S., Gauvreau K. (2005). Late Gestation Cerebellar Growth Is Rapid and Impeded by Premature Birth. *Pediatrics*.

[48] Volpe J. J. (2009). Cerebellum of the Premature Infant: Rapidly Developing, Vulnerable, Clinically Important. *Journal of Child Neurology*.

[49] Pires J. M., Foresti M. L., Silva C. S. (2020). Lipopolysaccharide-Induced Systemic Inflammation in the Neonatal Period Increases Microglial Density and Oxidative Stress in the Cerebellum of Adult Rats. *Frontiers in Cellular Neuroscience*.

[50] Stoessel M. B., Majewska A. K. (2021). Little Cells of the Little Brain: Microglia in Cerebellar Development and Function. *Trends in Neurosciences*.

[51] Iskusnykh I. Y., Chizhikov V. V. (2022). Cerebellar Development After Preterm Birth. *Frontiers in Cell and Developmental Biology*.

[52] Karst H., Berger S., Turiault M., Tronche F., Schütz G., Joëls M. (2005). Mineralocorticoid Receptors Are Indispensable for Nongenomic Modulation of Hippocampal Glutamate Transmission by Corticosterone. *Proceedings of the National Academy of Sciences*.

[53] Shishkina G. T., Kalinina T. S., Lanshakov D. A. (2023). Genes Involved by Dexamethasone in Prevention of Long-Term Memory Impairment Caused by Lipopolysaccharide-Induced Neuroinflammation. *Biomedicines*.

[54] Maggio N., Shavit-Stein E., Dori A., Blatt I., Chapman J. (2013). Prolonged Systemic Inflammation Persistently Modifies Synaptic Plasticity in the Hippocampus: Modulation by the Stress Hormones. *Frontiers in Molecular Neuroscience*.

[55] Koning A. C. A. M., Buurstede J. C., van Weert L. T. C. M., Meijer O. C. (2019). Glucocorticoid and Mineralocorticoid Receptors in the Brain: A Transcriptional Perspective. *Journal of the Endocrine Society*.

[56] Meijer O. C., de Kloet E. R. (2017). A Refill for the Brain Mineralocorticoid Receptor: The Benefit of Cortisol Add-On to Dexamethasone Therapy. *Endocrinology*.

[57] Rivers C. A., Rogers M. F., Stubbs F. E., Conway-Campbell B. L., Lightman S. L., Pooley J. R. (2019). Glucocorticoid Receptor–Tethered Mineralocorticoid Receptors Increase Glucocorticoid-Induced Transcriptional Responses. *Endocrinology*.

[58] Ahn J.-H., Lee H. J., Lee K. (2021). Effects of Lipopolysaccharide on Oligodendrocyte Differentiation at Different Developmental Stages: An In Vitro Study. *Journal of Korean Medical Science*.

[59] Pang Y., Cai Z., Rhodes P. G. (2003). Disturbance of Oligodendrocyte Development, Hypomyelination and White Matter Injury in the Neonatal Rat Brain After Intracerebral Injection of Lipopolysaccharide. *Developmental Brain Research*.

[60] Rousset C. I., Chalon S., Cantagrel S. (2006). Maternal Exposure to LPS Induces Hypomyelination in the Internal Capsule and Programmed Cell Death in the Deep Gray Matter in Newborn Rats. *Pediatric Research*.

[61] McArthur S., Pienaar I. S., Siddiqi S. M., Gillies G. E. (2016). Sex-Specific Disruption of Murine Midbrain Astrocytic and Dopaminergic Developmental Trajectories Following Antenatal GC Treatment. *Brain Structure and Function*.

[62] Van Steenwinckel J., Bokobza C., Laforge M. (2024). Key Roles of Glial Cells in the Encephalopathy of Prematurity. *Glia*.

