# Supplementary material for: Lipopolysaccharide Sensitizes Steroid-Induced Brain Injury in Neonatal Rat Pups
Source: Mediators Inflamm. 2025 Nov 20;2025:8285898. doi: 10.1155/mi/8285898 (PMC12660621; doi:10.1155/mi/8285898)
Supplement: Supporting Information 1 — Figure S1. The effect of single postnatal LPS administration on the rat pup lung. Representative H&E images of rat lungs harvested at postnatal Day 5 (P5). n = 3 in each group. Figure S2. Quantitative results of synaptophysin mRNA levels in various brain regions. Data are expressed as mean ± SD. n = 6; in certain cases, it is reduced to n = 5 due to the exclusion of outliers. Two-way ANOVA followed by Bonferroni's multiple comparisons was used to analyze differences among treatment (Sal and LPS) and drug (Sal, HC, and Dex). The details of statistical analysis are described in Supporting Information 3: Table S2. In the cerebellum, synaptophysin mRNA level was significantly higher in LPS-Sal group compared with Sal-Sal group. Figure S3. Quantitative results of PSD95 mRNA levels in various brain regions. Data are expressed as mean ± SD. n = 6; in certain cases, it is reduced to n = 5 due to the exclusion of outliers. Two-way ANOVA followed by Bonferroni's multiple comparisons was used to analyze differences among treatment (Sal and LPS) and drug (Sal, HC, and Dex). The details of statistical analysis are described in Supporting Information 3: Table S2. Figure S4. Body weight and brain weight by LPS vs. normal saline control and by sex. Data were expressed as mean ± SD. Three-way ANOVA followed by Bonferroni's multiple comparisons was used to analyze differences among treatment (Sal and LPS), drug (Sal, HC, and Dex) and sex (male and female). Sal-Sal (n = 8; male: 4, female: 4), Sal-HC (n = 8; male: 2, female: 6), Sal-Dex (n = 8; male: 5, female: 3), LPS-Sal (n = 8; male: 4, female: 4), LPS-HC (n = 8; male: 4, female: 4), and LPS-Dex (n = 8; male: 4, female: 4). The details of statistical analysis are described in Supporting Information 3: Table S2. Figure S5. Quantification of TUNEL stain in different brain regions, by LPS vs. normal saline control and by sex. Data were expressed as mean ± SD. Three-way ANOVA followed by Bonferroni's multiple comparisons was used to an [file 8285898.f1.docx]

**
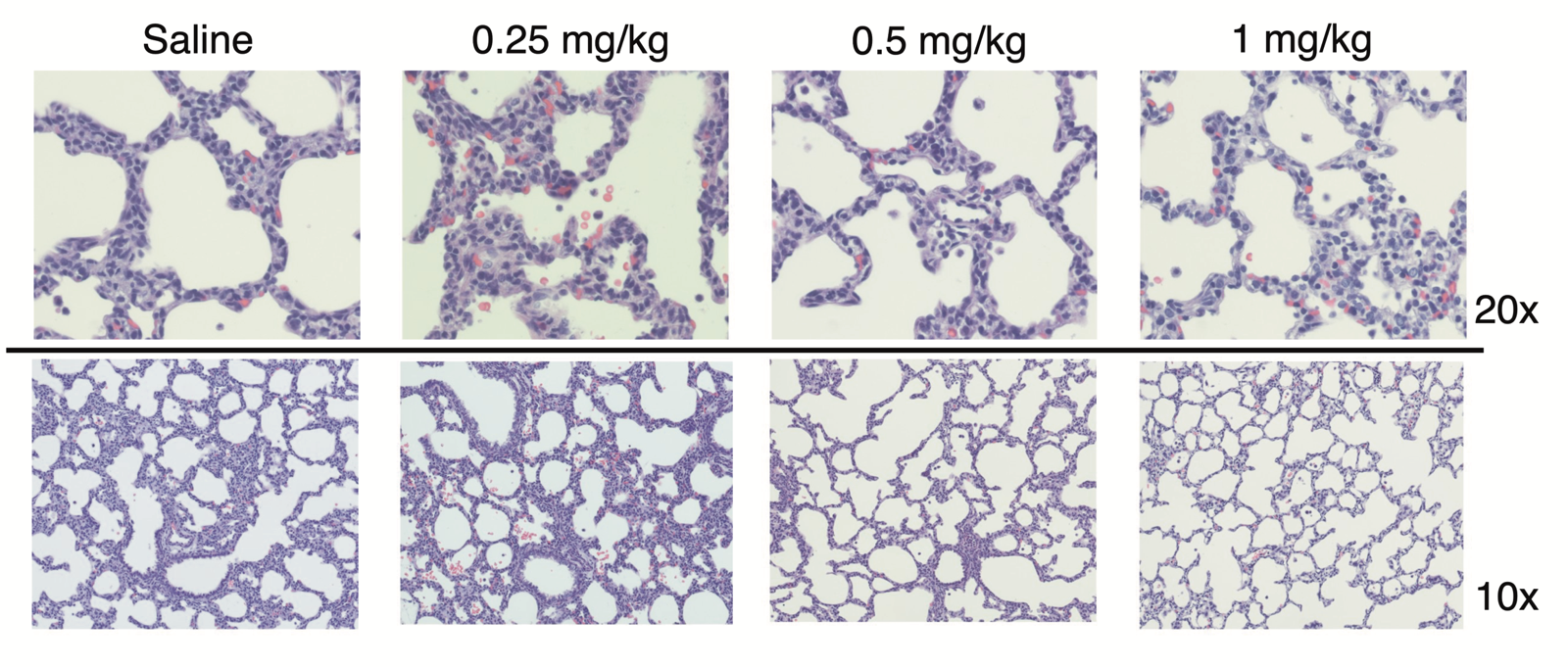
**

**Figure S1. The effect of single postnatal LPS administration on the rat pup lung.**Representative H&E images of rat lungs harvested at postnatal day 5 (P5). n = 3 in each group.


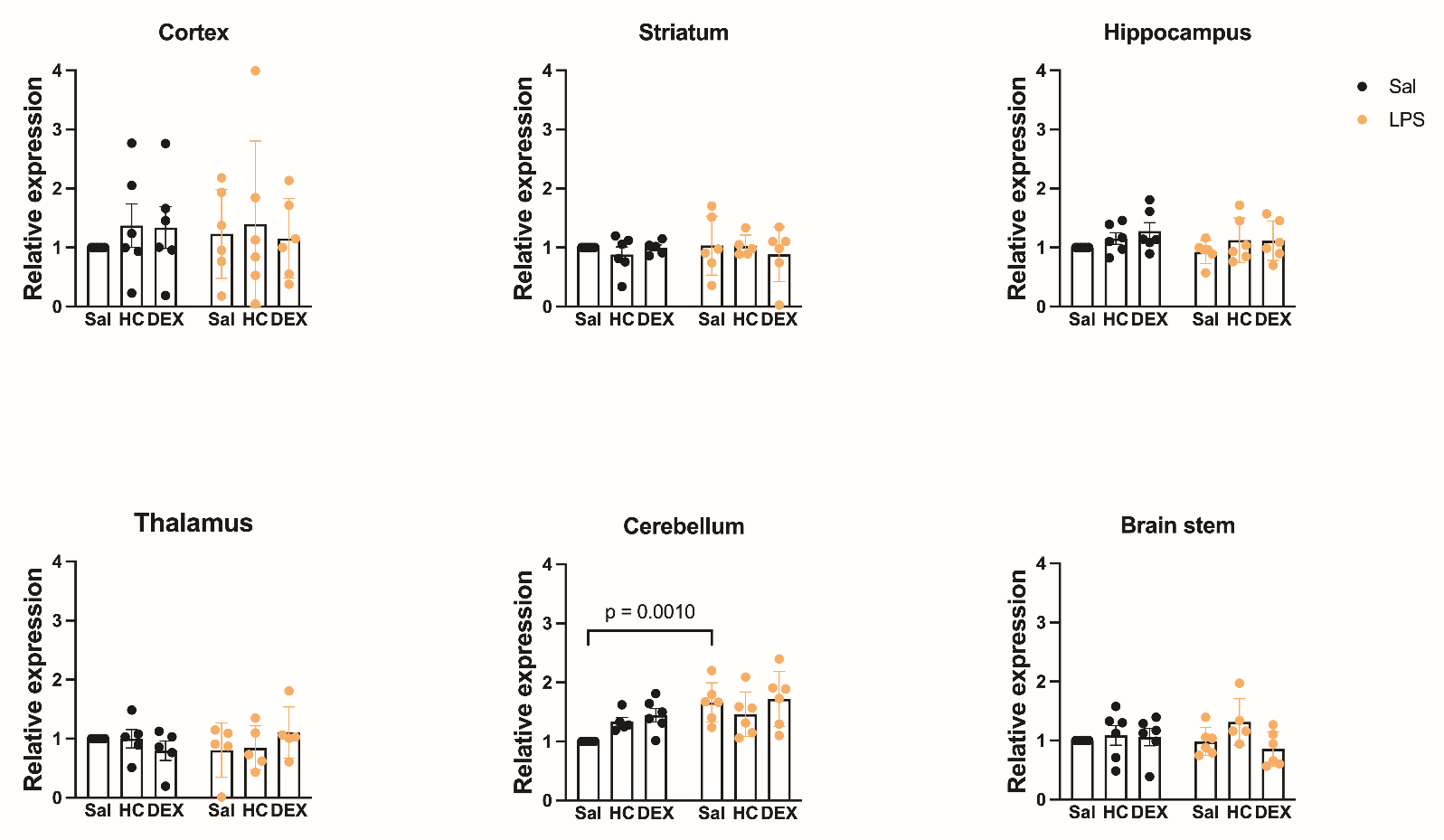


**Figure S2. Quantitative results of synaptophysin mRNA levels in various brain regions.** Data are expressed as mean ± SD. n = 6; in certain cases, it is reduced to n = 5 due to the exclusion of outliers. Two-way ANOVA followed by Bonferroni’s multiple comparisons was used to analyze differences among treatment (Sal and LPS) and drug (Sal, HC and Dex). The details of statistical analysis are described in Table S2. In the cerebellum, synaptophysin mRNA level was significantly higher in LPS-Sal group compared with Sal-Sal group.


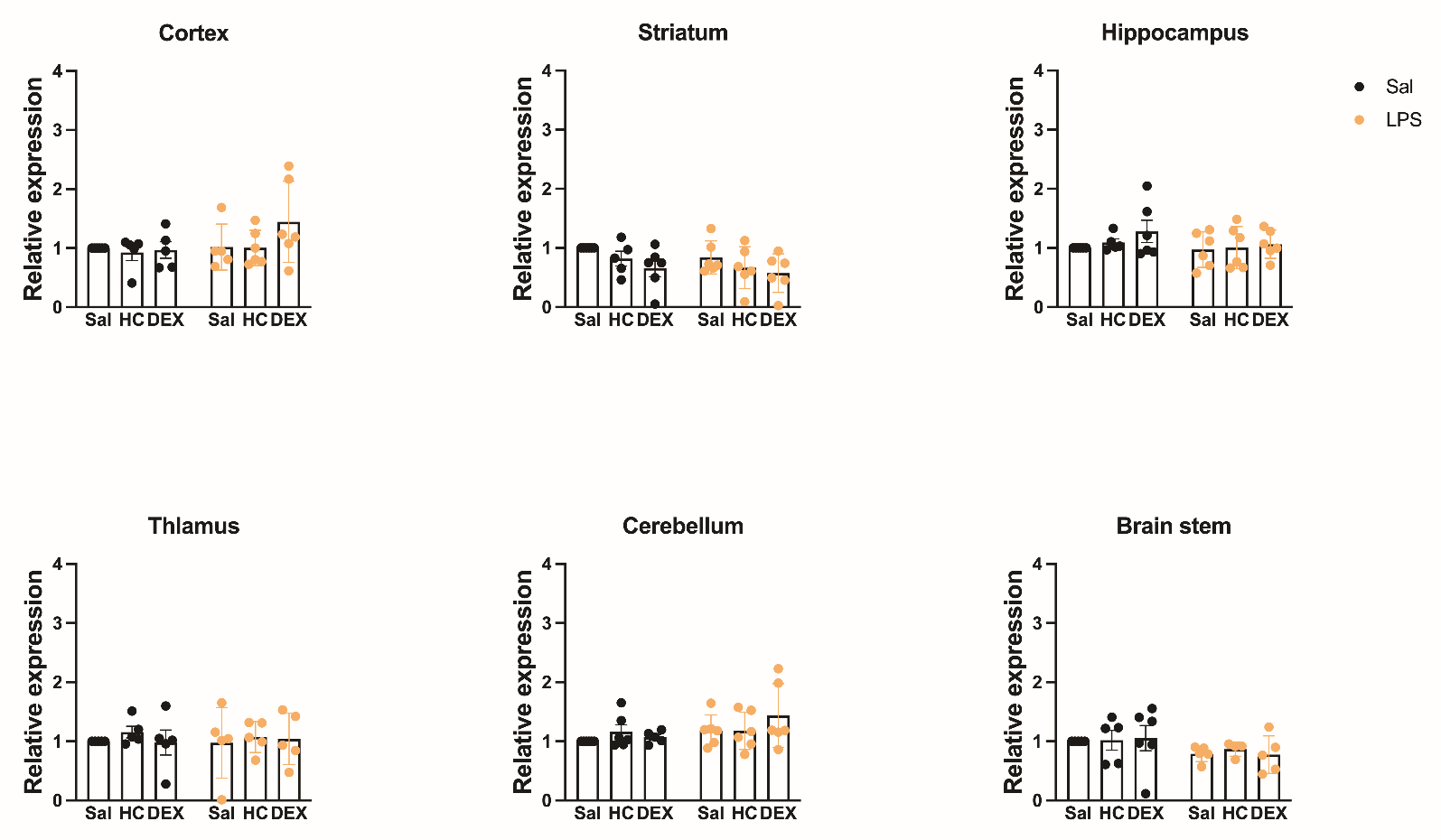


**Figure S3. Quantitative results of PSD95 mRNA levels in various brain regions.** Data are expressed as mean ± SD. n = 6; in certain cases, it is reduced to n = 5 due to the exclusion of outliers. Two-way ANOVA followed by Bonferroni’s multiple comparisons was used to analyze differences among treatment (Sal and LPS) and drug (Sal, HC and Dex). The details of statistical analysis are described in Table S2.


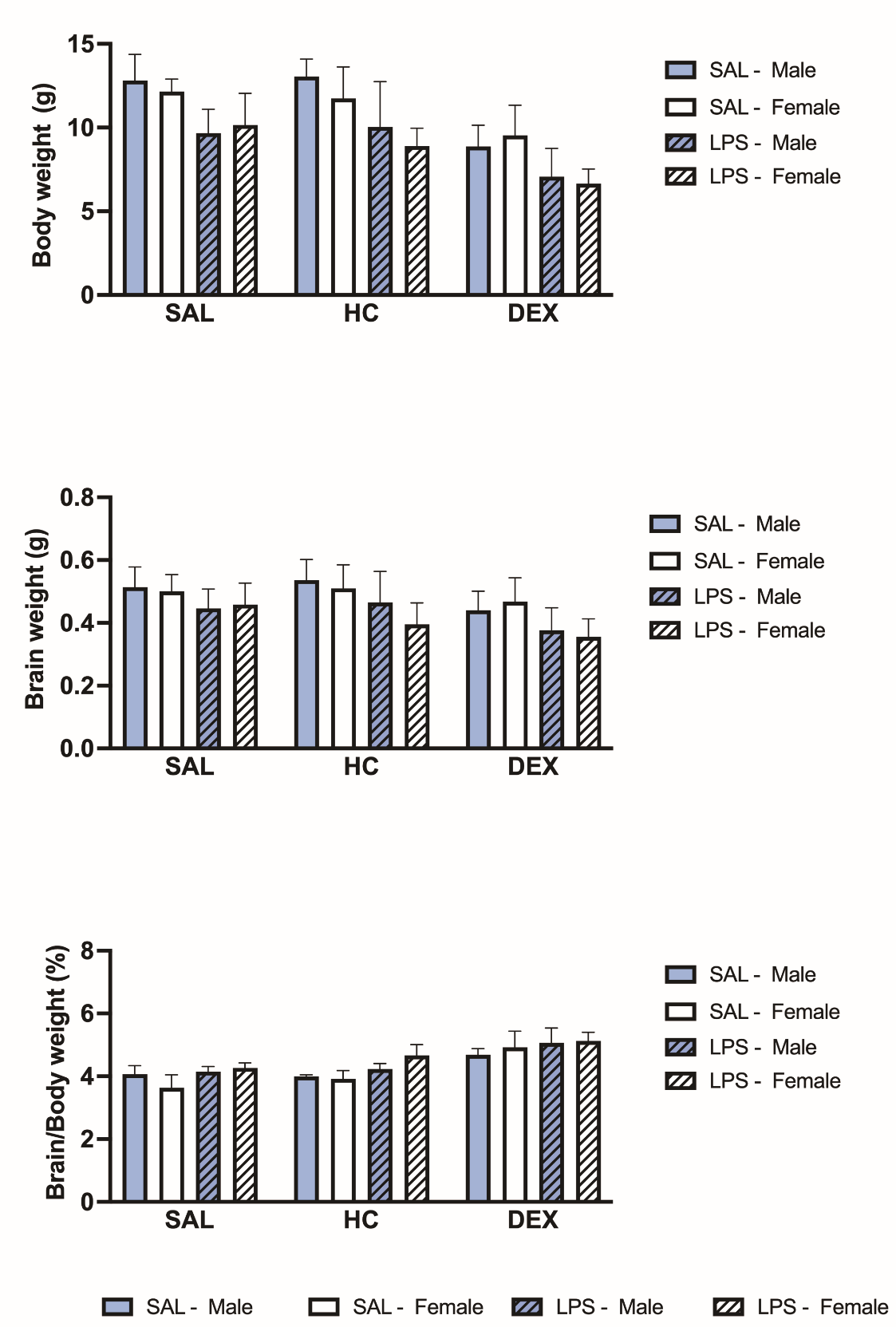


**Figure S4. Body weight and brain weight by LPS vs. normal saline control and by sex.** Data were expressed as mean ± SD. Three-way ANOVA followed by Bonferroni’s multiple comparisons was used to analyze differences among treatment (Sal and LPS), drug (Sal, HC and Dex) and sex (male and female). Sal-Sal (n = 8; male: 4, female: 4), Sal-HC (n = 8; male: 2, female: 6), Sal-Dex (n = 8; male: 5, female: 3), LPS-Sal (n = 8; male: 4, female: 4), LPS-HC (n = 8; male: 4, female: 4), and LPS-Dex (n = 8; male: 4, female: 4). The details of statistical analysis are described in Table S2.


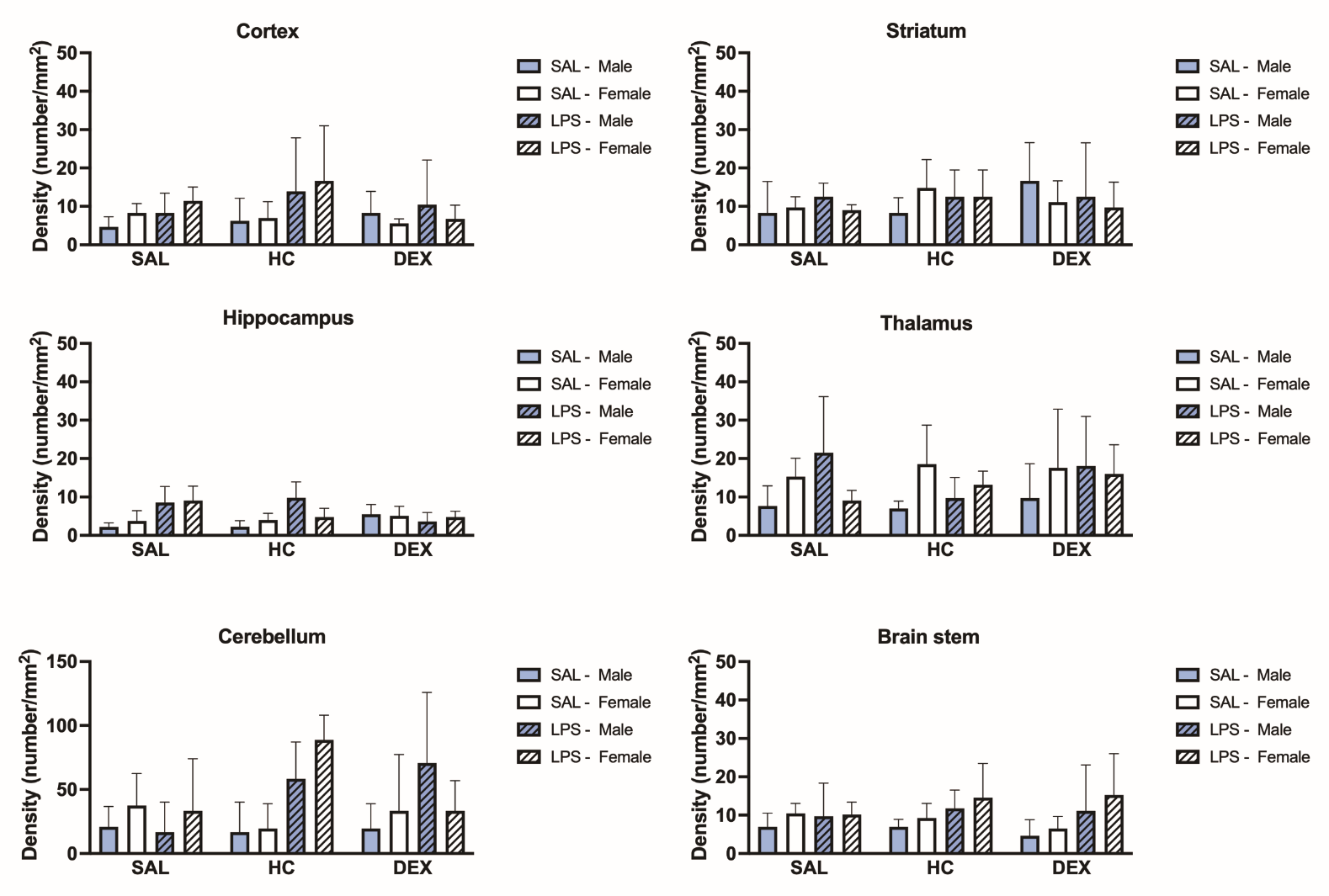


**Figure S5. Quantification of TUNEL stain in different brain regions, by LPS vs normal saline control and by sex.** Data were expressed as mean ± SD. Three-way ANOVA followed by Bonferroni’s multiple comparisons was used to analyze differences among treatment (Sal and LPS), drug (Sal, HC and Dex) and sex (male and female). Sal-Sal (n = 8; male: 4, female: 4), Sal-HC (n = 8; male: 2, female: 6), Sal-Dex (n = 8; male: 5, female: 3), LPS-Sal (n = 8; male: 4, female: 4), LPS-HC (n = 8; male: 4, female: 4), and LPS-Dex (n = 8; male: 4, female: 4). The details of statistical analysis are described in Table S2.


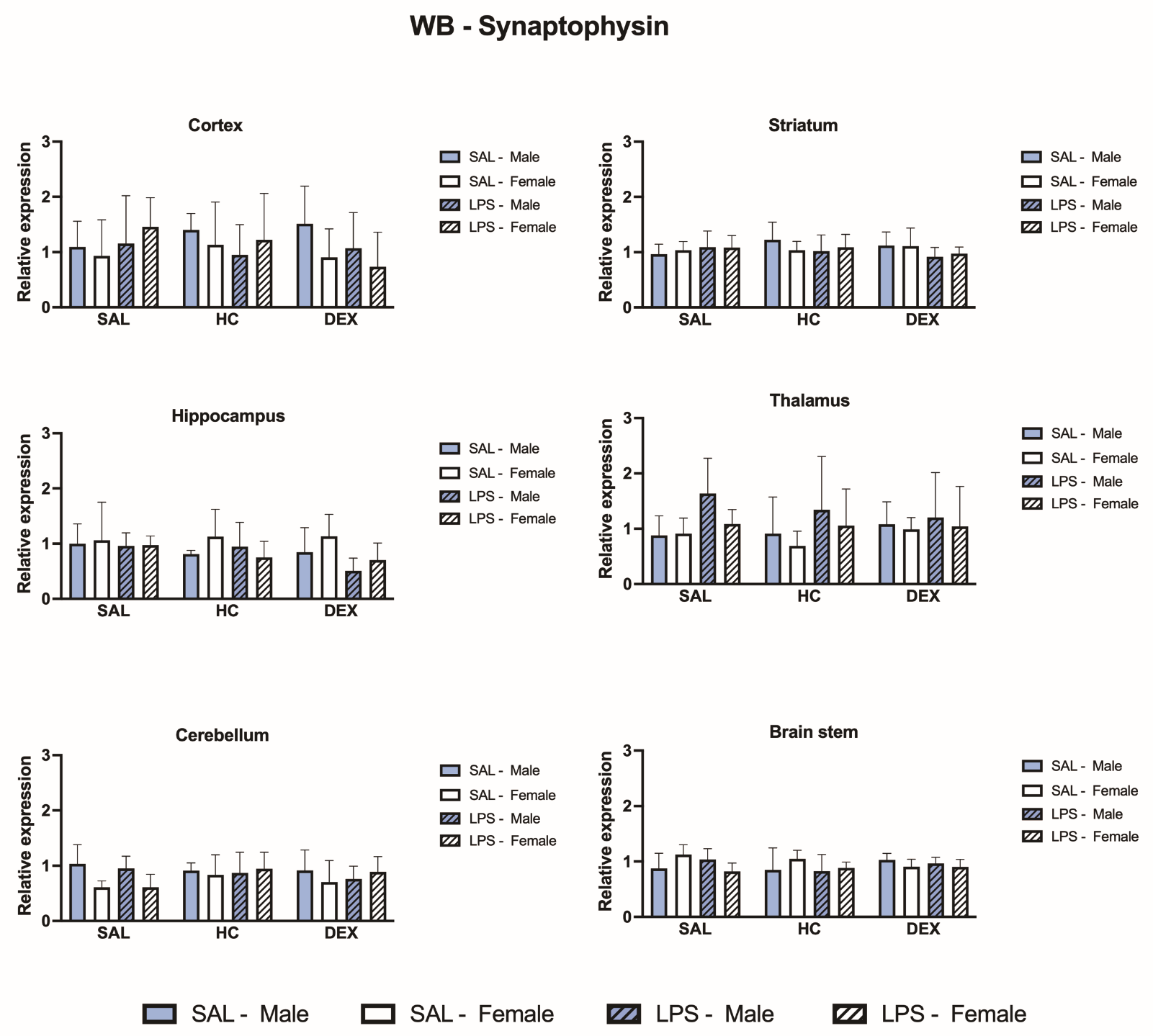


**Figure S6. Quantification of synaptophysin protein level (relative to control) in different brain regions, by LPS vs normal saline control and by sex.** Data were expressed as mean ± SD. Three-way ANOVA followed by Bonferroni’s multiple comparisons was used to analyze differences among treatment (Sal and LPS), drug (Sal, HC and Dex) and sex (male and female). Sal-Sal (n = 8; male: 4, female: 4), Sal-HC (n = 8; male: 2, female: 6), Sal-Dex (n = 8; male: 5, female: 3), LPS-Sal (n = 8; male: 4, female: 4), LPS-HC (n = 8; male: 4, female: 4), and LPS-Dex (n = 8; male: 4, female: 4). The details of statistical analysis are described in Table S2.


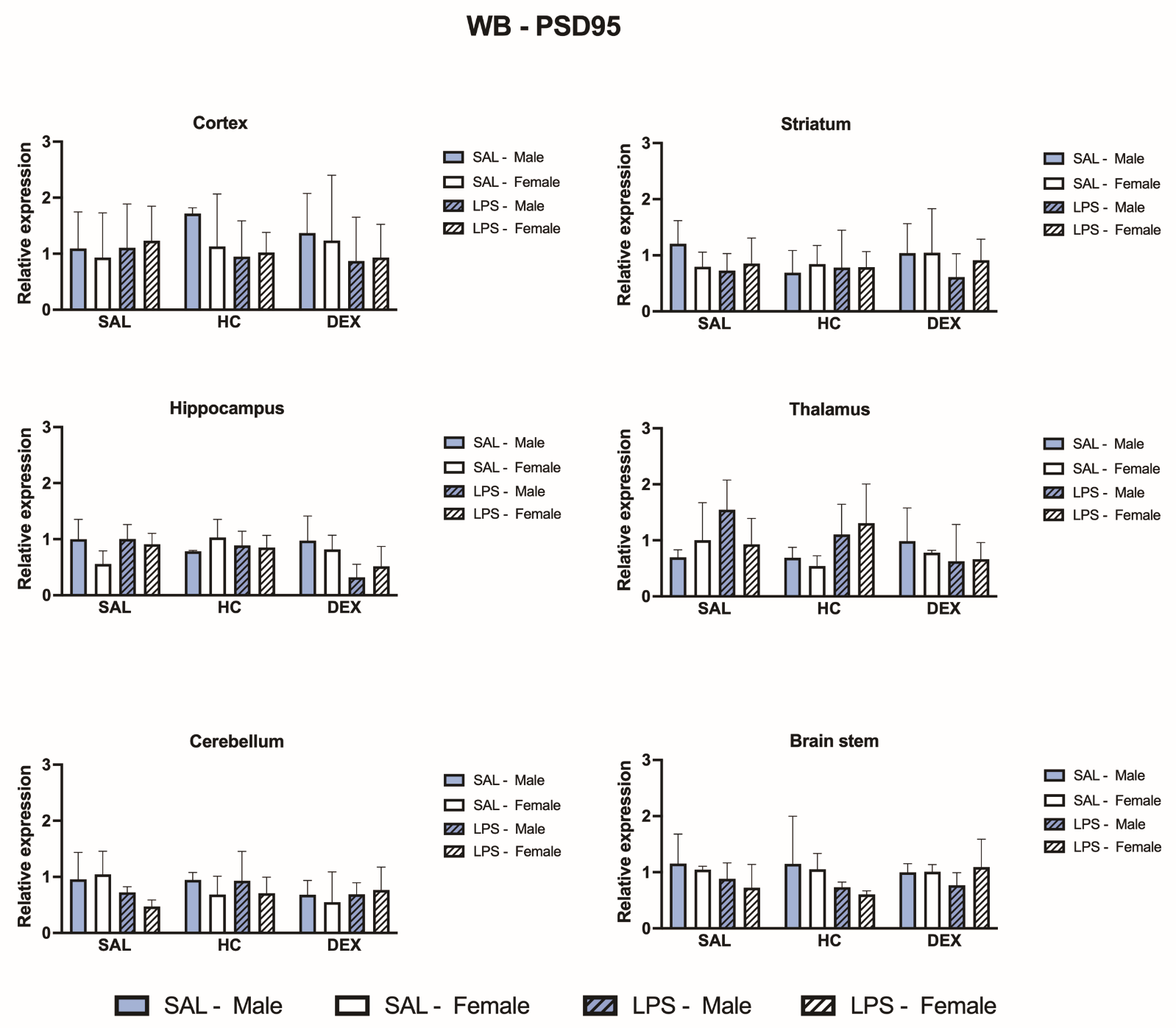


**Figure S7. Quantification of PSD95 protein level (relative to control) in different brain regions, by LPS vs normal saline control and by sex.** Data were expressed as mean ± SD. Three-way ANOVA followed by Bonferroni’s multiple comparisons was used to analyze differences among treatment (Sal and LPS), drug (Sal, HC and Dex) and sex (male and female). Sal-Sal (n = 8; male: 4, female: 4), Sal-HC (n = 8; male: 2, female: 6), Sal-Dex (n = 8; male: 5, female: 3), LPS-Sal (n = 8; male: 4, female: 4), LPS-HC (n = 8; male: 4, female: 4), and LPS-Dex (n = 8; male: 4, female: 4). The details of statistical analysis are described in Table S2.

**
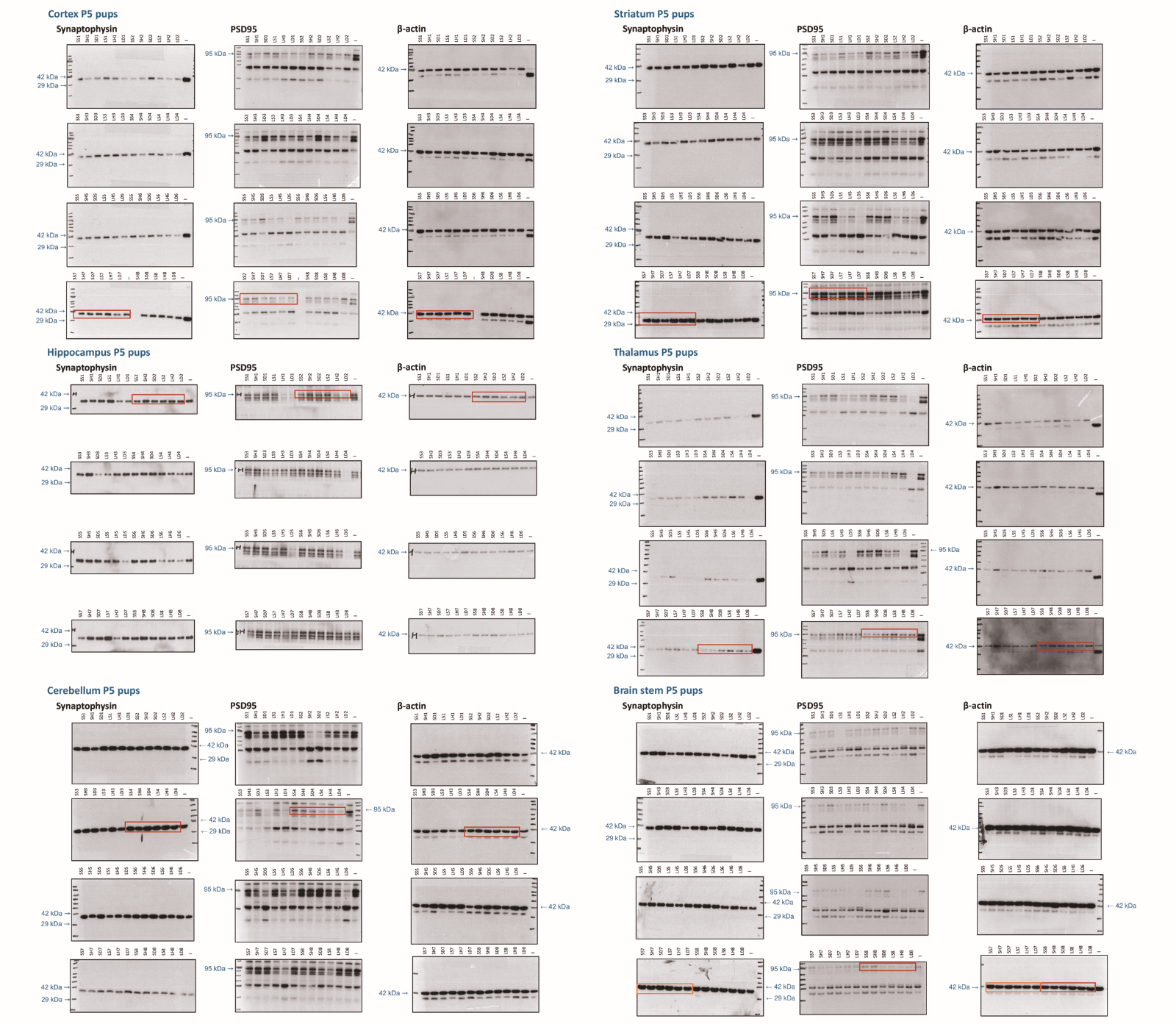
**

**Figure S8. Whole gel images of western blots.** This image shows the unedited western blot images of Synaptophysin, PSD95, and β-actin across all six brain regions. SS: Sal-Sal; SH: Sal-HC; SD: Sal-Dex; LS: LPS-Sal; LH: LPS-HC; LD: LPS-Dex.


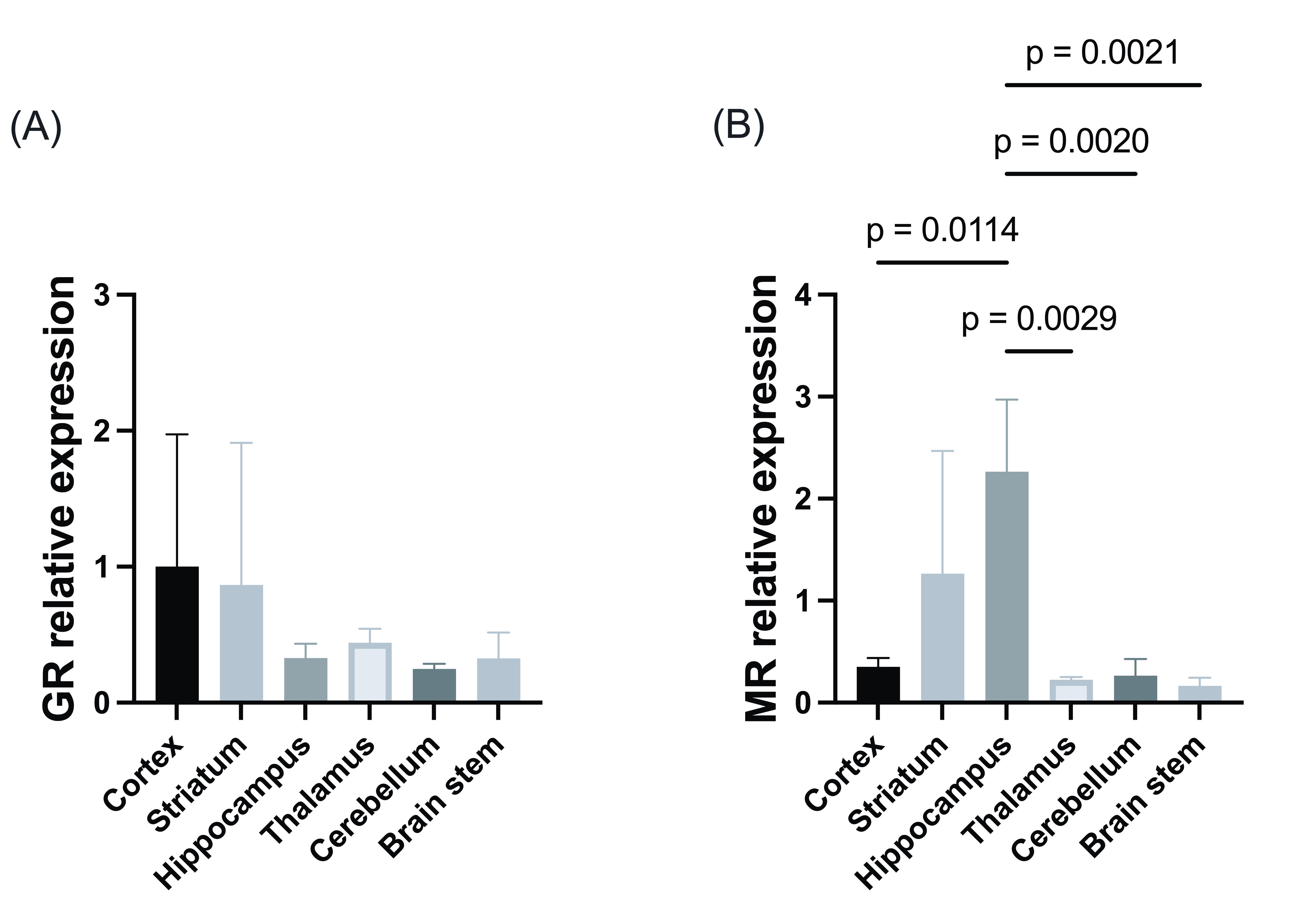


**Figure S9. Comparative results of glucocorticoid receptor (GR) and mineralocorticoid (MR) mRNA levels in various brain regions in saline control animals.** Data are expressed as mean ± SD. n = 5; in certain cases, it is reduced to n = 4 due to the exclusion of outliers.
