# Supplementary material for: Lipopolysaccharide Sensitizes Steroid-Induced Brain Injury in Neonatal Rat Pups
Source: Mediators Inflamm. 2025 Nov 20;2025:8285898. doi: 10.1155/mi/8285898 (PMC12660621; doi:10.1155/mi/8285898)
Supplement: Supporting Information 2 — Table S1. Primer sequences for RT-qPCR. [file 8285898.f2.docx]

| Synaptophysin | Forward | 5’-ACTACTCCTCGTCGGCTGAA-3’ |
| --- | --- | --- |
| Synaptophysin | Reverse | 5’-GTTCCTTGCATGTGTTCCCT-3’ |
| PSD95 | Forward | 5’-TCCAGTCTGTGCGAGAGGTAGC-3’ |
| PSD95 | Reverse | 5’-GGACGGATGAAGATGGCGATGG-3’ |
| GAPDH | Forward | 5’- CCATCTTCCAGGAGCGAGATC -3’ |
| GAPDH | Reverse | 5’- GCCTTCTCCATGGTGGTGAA -3’ |
| MR | Forward | 5’- AGCTCTTCTGTTAGCAGCCCGCTG -3’ |
| MR | Reverse | 5’- CTGAAGTGGCATAGCTGAAGGCAT -3’ |
| GR | Forward | 5’- TGCAAACCTCAATAGGTCGACCAG -3’ |
| GR | Reverse | 5’- TAAACTGGGCCCAGTTTCTCTTGC -3’ |

**Table S1. Primer Sequences for RT-qPCR.**
